# Identification and description of patients with multisystem inflammatory syndrome in adults associated with SARS-CoV-2 infection using the Premier Healthcare Database

**DOI:** 10.1017/S0950268822000024

**Published:** 2022-01-17

**Authors:** Jennifer DeCuir, James Baggs, Michael Melgar, Pragna Patel, Karen K. Wong, Noah G. Schwartz, Sapna Bamrah Morris, Shana Godfred-Cato, Ermias D. Belay

**Affiliations:** 1 COVID-19 Response Team, Centers for Disease Control and Prevention, Atlanta, GA, USA; 2 Epidemic Intelligence Service, Centers for Disease Control and Prevention, Atlanta, GA, USA; 3 U.S. Public Health Service, Rockville, MD, USA

**Keywords:** Multisystem inflammatory syndrome in adults, MIS-A, post-COVID conditions, SARS-CoV-2, COVID-19

## Abstract

Multisystem inflammatory syndrome in adults (MIS-A) is a hyperinflammatory illness related to severe acute respiratory syndrome coronavirus 2 (SARS-CoV-2) infection. The characteristics of patients with this syndrome and the frequency with which it occurs among patients hospitalised after SARS-CoV-2 infection are unclear. Using the Centers for Disease Control and Prevention case definition for MIS-A, we created ICD-10-CM code and laboratory criteria to identify potential MIS-A patients in the Premier Healthcare Database Special COVID-19 Release, a database containing patient-level information on hospital discharges across the United States. Modified MIS-A criteria were applied to hospitalisations with discharge from March to December 2020. The proportion of hospitalisations meeting electronic health record criteria for MIS-A and descriptive statistics for patients in the potential MIS-A cohort were calculated. Of 34 515 SARS-CoV-2-related hospitalisations with complete clinical and laboratory data, 53 met modified criteria for MIS-A (0.15%). The median age was 62 years (IQR 52–74). Most patients met the severe cardiac illness criterion through either myocarditis (66.0%) or new-onset heart failure (35.8%). A total of 79.2% of patients required ICU admission, while 43.4% of patients in the cohort died. MIS-A appears to be a rare but severe outcome of SARS-CoV-2 infection. Additional studies are needed to investigate how this syndrome differs from severe coronavirus disease 2019 (COVID-19) in adults.

## Introduction

As the coronavirus disease 2019 (COVID-19) pandemic has progressed, our understanding of the natural history of infection due to severe acute respiratory syndrome coronavirus 2 (SARS-CoV-2) has evolved. Accumulating evidence has identified multiple phenotypes of disease that appear to be associated with SARS-CoV-2 infection, including acute COVID-19, multisystem inflammatory syndrome (MIS), and other post-COVID conditions [1, 2]. MIS is a post-acute hyperinflammatory illness occurring 2–6 weeks after SARS-CoV-2 infection [3, 4]. Although most research to date has focused on multisystem inflammatory syndrome in children (MIS-C), numerous cases of multisystem inflammatory syndrome in adults (MIS-A) have been reported [4–7].

Published case reports of MIS-A describe younger patients with few comorbid conditions [5, 8]. The syndrome is characterised by fever and prominent cardiovascular manifestations, including cardiac dysfunction with newly reduced left ventricular ejection fraction, myocarditis, cardiogenic shock, and vasoplegic shock [5–7]. Patients may also present with mucocutaneous manifestations similar to those observed in Kawasaki disease, including rash, non-purulent conjunctivitis, and oral mucosal changes [4, 9, 10]. Gastrointestinal symptoms, such as diarrhoea, abdominal pain, and vomiting, are also common [5]. Laboratory testing is notable for elevated inflammatory and cardiac markers [4]. Because MIS-A occurs in the post-acute period, host clearance of SARS-CoV-2 infection and the concomitant development of antibodies to SARS-CoV-2 may have already occurred. Therefore, serologic testing for antibodies to SARS-CoV-2 is often positive, while RT-PCR testing may be negative [4]. Patients with MIS-A often require intensive care and vasopressor or inotropic support. Other agents used in the management of MIS-A include corticosteroids, intravenous immunoglobulin (IVIG), and tocilizumab, although the efficacy of these therapies has not been evaluated in clinical trials. In severe cases, patients have been treated with mechanical circulatory support and extracorporeal membrane oxygenation (ECMO) [6, 7, 11]. Regardless of age, patients with MIS-A may be critically ill on presentation, and mortality related to MIS-A has been reported [5].

**Fig. 1.**
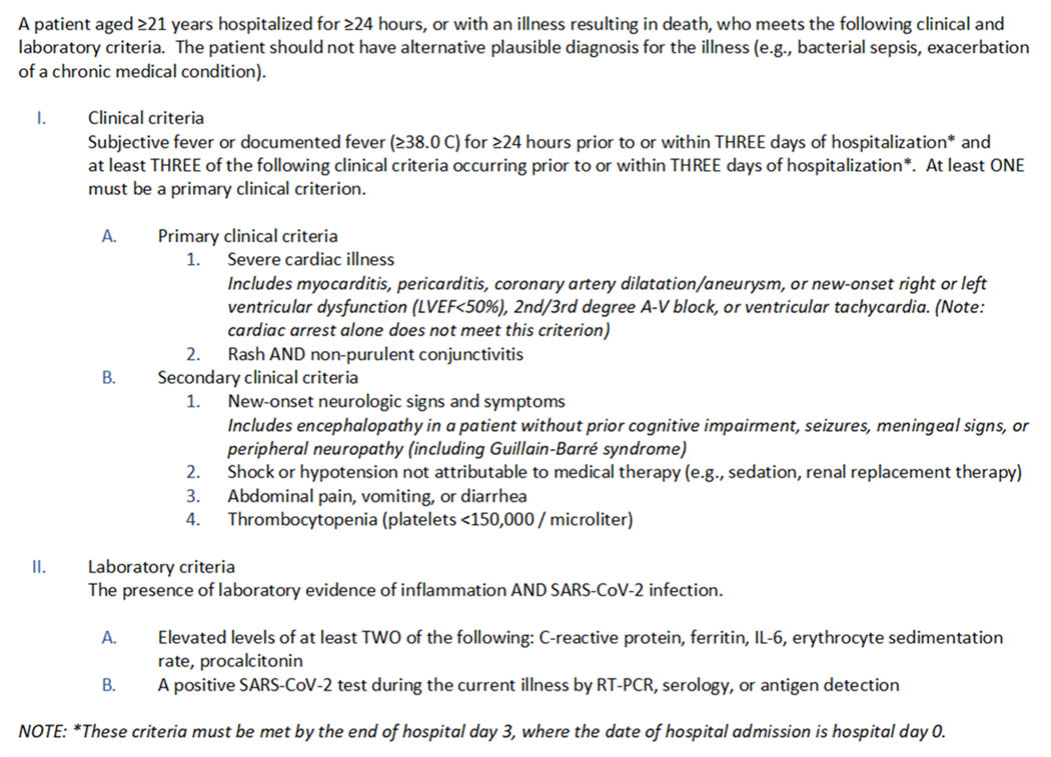
Centers for Disease Control and Prevention case definition for multisystem inflammatory syndrome in adults (MIS-A) associated with SARS-CoV-2 infection [12].

The Centers for Disease Control and Prevention (CDC) published a case definition for MIS-A on 11 May 2021 to improve recognition and surveillance (Fig. 1) [12]; however, many unanswered questions remain. The objectives of this investigation were to determine what proportion of SARS-CoV-2-related hospitalisations might meet electronic health record criteria for MIS-A and to describe their demographic, clinical, and laboratory characteristics. To investigate these objectives, we applied a modified version of the CDC case definition for MIS-A to a large database of inpatient hospital discharges in the United States.

## Methods

This analysis was conducted using data from the Premier Healthcare Database Special COVID-19 Release (PHD-SR, release date: 24 March 2021), an all-payer database containing patient-level information on inpatient discharges from over 800 non-governmental, community, and teaching hospitals located in geographically diverse areas of the United States. Administrative discharge data included in the PHD-SR represent approximately 25% of annual inpatient admissions in the United States [13].

The current analysis was limited to discharges occurring from March to December 2020. This study period was chosen because it predates the *International Classification of Diseases, Tenth Revision, Clinical Modification* (ICD-10-CM) diagnosis code for MIS-A, which was introduced in January 2021 [12]. Hospitalisations consistent with MIS-A were identified in the PHD-SR using a set of criteria adapted from the CDC case definition for MIS-A. ICD-10-CM codes and laboratory data used to define criteria are described in detail in the Supplementary Material. In brief, hospitalisations were required to meet the following criteria for inclusion: (1) Age ⩾ 21 years; (2) SARS-CoV-2-related hospitalisation; (3) Laboratory evidence of severe inflammation, as evidenced by elevated levels of ⩾2 of the following on hospital day 0–3: C-reactive protein (CRP), erythrocyte sedimentation rate (ESR), ferritin, interleukin-6 (IL-6), or procalcitonin; and (4) The presence of severe cardiac illness plus ⩾1 of the following clinical criteria: mucocutaneous involvement (rash or conjunctivitis), shock/hypotension, gastrointestinal involvement (abdominal pain, vomiting, diarrhoea, or gastroenteritis), or thrombocytopenia. Severe cardiac illness was defined as the presence of new-onset heart failure, myocarditis, pericarditis, or coronary artery aneurysm.

Hospitalisations were excluded from the cohort if the patient had a history of chronic heart failure or if the hospitalisation had a diagnosis code for an alternative cause of new-onset heart failure (e.g. ST-elevation MI, ischaemic cardiomyopathy, hypertrophic cardiomyopathy). Hospitalisations were also excluded if they had a diagnosis code for sepsis due to a pathogen other than SARS-CoV-2. To determine which patients had a history of chronic heart failure, the Elixhauser algorithm was applied to hospital encounter data from the Premier Healthcare Database dating from 1 January 2019 up to, but not including, each patient's first SARS-CoV-2-related hospitalisation [14]. Chronic heart failure was defined as either (1) a history of congestive heart failure (CHF) according to the Elixhauser algorithm, or (2) a diagnosis code from the SARS-CoV-2-related hospitalisation indicative of chronic heart failure. ICD-10-CM codes used to define chronic heart failure, alternative causes of new-onset heart failure, and sepsis due to an alternative pathogen are provided in the Supplementary Material.

Although the criteria used to define the potential MIS-A cohort were modelled on the CDC case definition for MIS-A, certain aspects of the case definition could not be applied due to missing or unavailable data. For example, data on fever and SARS-CoV-2 testing were available for too few hospitalisations to be used to define the cohort. In addition, identification of clinical criteria was limited to the use of ICD-10-CM diagnosis codes, which gave no indication as to the timing of clinical manifestations during the hospitalisation. For this reason, the requirement that clinical criteria occur during the first 3 days of hospitalisation in the CDC case definition was modified to requiring at least two elevated inflammatory markers in the first 3 days of hospitalisation for our analysis. Our criteria were also modified to maintain both specificity and sensitivity for identifying MIS-A. Because rash is a non-specific diagnosis with multiple aetiologies aside from MIS-A, mucocutaneous manifestations were operationalised as a secondary clinical criterion as opposed to a primary clinical criterion. Similarly, cardiac arrhythmias and neurologic manifestations were not included in the criteria. To account for underreporting of signs and symptoms such as rash and abdominal pain by ICD-10-CM diagnosis codes, the number of clinical criteria required for inclusion in the potential MIS-A cohort was lowered from three to two for our analysis.

The proportion of hospitalisations meeting modified MIS-A criteria was calculated by dividing the number of hospitalisations meeting modified MIS-A criteria by the number of hospitalisations in the PHD-SR database among patients aged ⩾21 years with at least two inflammatory markers available to which the full MIS-A criteria could be applied. Descriptive statistics were calculated for demographic, clinical and laboratory characteristics of hospitalisations in the cohort. ICD-10-CM diagnosis codes used to determine respiratory involvement and cardiovascular circulatory support are provided in the Supplementary Material. Descriptive statistics are presented for the entire cohort and stratified by age less than 50 years and greater than or equal to 50 years. Age 50 years was chosen as the cutoff for this stratification because few MIS-A cases have been reported among patients greater than 50 years old, and patients in this older age group may have met modified MIS-A criteria through alternative diagnoses, such as severe COVID-19 [5, 8, 15, 16]. Fisher's exact tests were used to compare categorical variables. Numeric variables with medians and interquartile ranges (IQR) were compared using the Wilcoxon rank-sum test. Analyses were performed in SAS (version 9.4; SAS Institute).

## Results

**Fig. 2.**
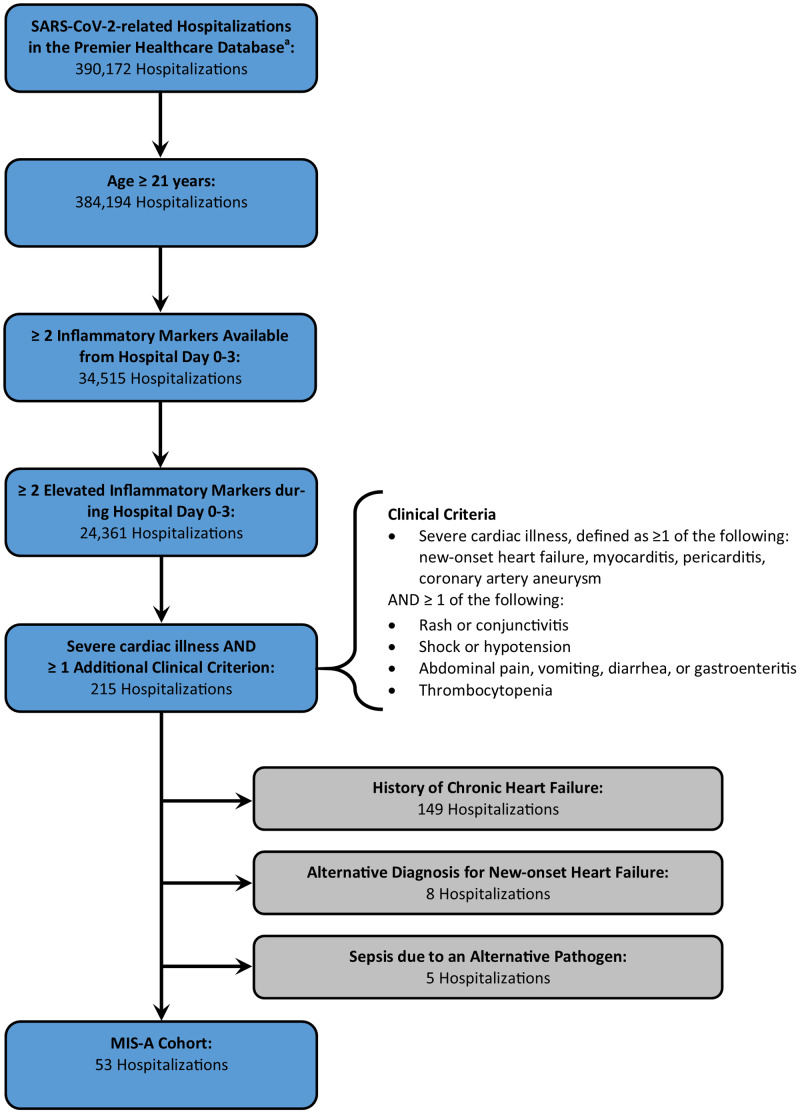
Defining the cohort meeting modified criteria for multisystem inflammatory syndrome in adults associated with SARS-CoV-2 infection in the Premier Healthcare Database Special COVID-19 Release. ^a^Includes hospitalisations with discharge from March to December 2020.

A total of 390 172 SARS-CoV-2-related hospitalisations were available from the PHD-SR with discharges during March–December 2020. Figure 2 describes how the number of hospitalisations remaining in the cohort decreased as modified MIS-A criteria were applied. Of note, laboratory data on inflammatory markers within the first 3 days of hospitalisation were available for 9.0% of SARS-CoV-2-related hospitalisations among patients ⩾21 years of age (34 515 hospitalisations). Hospitalisations with available laboratory data were more likely to require ICU admission (48.8% *vs.* 44.4%, *P* < 0.0001) and less likely to end in death (10.5% *vs.* 13.7%, *P* < 0.0001) when compared to those without laboratory data. Median age (64 years *vs.* 66 years) and length of stay (5 days *vs.* 5 days) were similar among hospitalisations with and without available laboratory data. The final cohort contained 53 hospitalisations, of which 12 occurred among patients less than 50 years of age. There were no patients with more than one hospitalisation in the final cohort. Of note, if three clinical criteria had been required for inclusion in the potential MIS-A cohort, the final cohort would have contained 24 hospitalisations, with 6 occurring among patients less than 50 years of age.

The proportion of SARS-CoV-2-related hospitalisations that met modified MIS-A criteria was 0.15% (53/34 515) among all patients, 0.17% among patients under 50 (12/7245), and 0.15% among patients greater than or equal to 50 years of age (41/27 270).

### Demographic, clinical, and laboratory characteristics among all patients

Among the 53 patients who met modified MIS-A criteria, the median age was 62 years (IQR 52–74 years), and the cohort was 56.6% male (Table 1). Most patients were non-Hispanic white (52.8%), followed by non-Hispanic black (18.9%), Hispanic (13.2%), Asian (7.5%), unknown race (5.7%), and other race (1.9%).

**Table 1.** Demographic characteristics and comorbidities of patients meeting modified criteria for multisystem inflammatory syndrome in adults with discharge during March–December 2020 in the Premier Healthcare Database Special COVID-19 Release

	All patients *N* = 53	Patients <50 years-old *N* = 12	Patients ⩾50 years-old *N* = 41	*P*-value
Demographics				
Age – median (IQR)	62 (52–74)	41 (32–44)	65 (58–75)	^a^
Male sex – *n* (%)	30 (56.6)	9 (75.0)	21 (51.2)	0.19
Race/ethnicity – *n* (%)				0.15^b^
Hispanic or Latino	7 (13.2)	3 (25.0)	4 (9.8)	
White, non-Hispanic	28 (52.8)	3 (25.0)	25 (61.0)	
Black, non-Hispanic	10 (18.9)	4 (33.3)	6 (14.6)	
Asian, non-Hispanic	4 (7.5)	1 (8.3)	3 (7.3)	
Other race, non-Hispanic	1 (1.9)	0 (0.0)	1 (2.4)	
Unknown race, non-Hispanic	3 (5.7)	1 (8.3)	2 (4.9)	
Chronic medical conditions – *n* (%)				
Obesity	11 (20.8)	4 (33.3)	7 (17.1)	0.24
Hypertension	26 (49.1)	2 (16.7)	24 (58.5)	0.02
Diabetes	16 (30.2)	2 (16.7)	14 (34.2)	0.31
Chronic pulmonary disease	17 (32.1)	4 (33.3)	13 (31.7)	1.00
Chronic kidney disease	7 (13.2)	0 (0.0)	7 (17.1)	0.33
Number of chronic medical conditions – median (IQR)	1 (1–2)	1 (0–1.5)	1 (1–2)	0.08

IQR, interquartile range.

a *P*-value not calculated.

b *P*-value represents comparison across all race/ethnicity categories.

By definition, all patients in the potential MIS-A cohort had a severe cardiac illness (Table 2). The next most common clinical criteria were shock or hypotension (77.4%), thrombocytopenia (52.8%), and gastrointestinal symptoms (18.9%). None of the patients in the cohort had reported mucocutaneous manifestations. Among the severe cardiac illnesses included in our analysis, myocarditis (66.0%) and new-onset heart failure (35.9%) were the most common, while pericarditis (5.7%) and coronary artery aneurysm (1.9%) were less common. The most common gastrointestinal symptom was diarrhoea (17.0%). One patient in the cohort had gastroenteritis (1.9%), and there were no patients with reported abdominal pain or vomiting. The median length of stay in the hospital was 7 days (IQR 3–16 days), with 79.3% of hospitalisations requiring ICU admission. A total of 23 patients in the cohort died (43.4%). Among the patients who died, the median age was 61 years (IQR 48–73 years) and 14 (60.9%) of the patients were male.

**Table 2.** Clinical characteristics of patients meeting modified criteria for multisystem inflammatory syndrome in adults with discharge during March–December 2020 in the Premier Healthcare Database Special COVID-19 Release

	All patients *N* = 53	Patients <50 years-old *N* = 12	Patients ⩾50 years-old *N* = 41	*P*-value
Modified MIS-A criteria – *n* (%)				
Severe cardiac illness	53 (100)	12 (100)	41 (100)	1.00
New-onset heart failure	19 (35.8)	6 (50.0)	13 (31.7)	0.31
Myocarditis	35 (66.0)	9 (75.0)	26 (63.4)	0.73
Pericarditis	3 (5.7)	1 (8.3)	2 (4.9)	0.55
Coronary artery aneurysm	1 (1.9)	0 (0.0)	1 (2.4)	1.00
Shock/Hypotension	41 (77.4)	8 (66.7)	33 (80.5)	0.43
Gastrointestinal	10 (18.9)	2 (16.7)	8 (19.5)	1.00
Abdominal pain	0 (0.0)	0 (0.0)	0 (0.0)	^a^
Vomiting	0 (0.0)	0 (0.0)	0 (0.0)	^a^
Diarrhoea	9 (17.0)	1 (8.3)	8 (19.5)	0.67
Gastroenteritis	1 (1.9)	1 (8.3)	0 (0.0)	0.23
Thrombocytopenia	28 (52.8)	8 (66.7)	20 (48.8)	0.34
Mucocutaneous	0 (0.0)	0 (0.0)	0 (0.0)	^a^
Rash	0 (0.0)	0 (0.0)	0 (0.0)	^a^
Conjunctivitis	0 (0.0)	0 (0.0)	0 (0.0)	^a^
Respiratory involvement – *n* (%)	47 (88.7)	10 (83.3)	37 (90.2)	0.61
Outcomes – median (IQR), *n* (%)				
Length of stay (days)	7 (3–16)	6.5 (3–12)	7 (4–16)	0.42
ICU admission	42 (79.2)	11 (91.7)	31 (75.6)	0.42
Died	23 (43.4)	6 (50.0)	17 (41.5)	0.74

MIS-A, multisystem inflammatory syndrome in adults; IQR, interquartile range; ICU, intensive care unit.

a No statistics were calculated because there were no patients in the potential MIS-A cohort with this sign or symptom.

Median CRP, ESR, ferritin, interleukin-6, and procalcitonin were all highly elevated (Table 3). Many patients in the cohort were treated with vasoactive medications (58.5%), and two received intra-aortic balloon pumps (Table 4). Many patients were treated with intravenous corticosteroids (73.6%) and tocilizumab (34.0%). One patient received IVIG (1.9%).

**Table 3.** Laboratory values of patients meeting modified criteria for multisystem inflammatory syndrome in adults with discharge during March–December 2020 in the Premier Healthcare Database Special COVID-19 Release

	All patients *N* = 53	Patients <50 years-old *N* = 12	Patients ⩾50 years-old *N* = 41	*P*-value
Blood cell counts^a^ – *n* (%)				
Leucocytosis	26 (53.1) *N* = 49	10 (83.3) *N* = 12	16 (43.2) *N* = 37	0.02
Neutrophilia	28 (58.3) *N* = 48	9 (90.0) *N* = 10	19 (50.0) *N* = 38	0.03
Lymphopenia	36 (76.6) *N* = 47	6 (75.0) *N* = 8	30 (76.9) *N* = 39	1.00
Anemia	40 (75.5) *N* = 53	9 (75.0) *N* = 12	31 (75.6) *N* = 41	1.00
Thrombocytopenia	26 (49.1) *N* = 53	7 (58.3) *N* = 12	19 (46.3) *N* = 41	0.53
Markers of inflammation – median (IQR)				
C-reactive protein (mg/dl)	14.1 (9.8–24.0) *N* = 50	22.3 (15.6–36.8) *N* = 12	11.3 (8.4–21.3) *N* = 38	0.03
Erythrocyte sedimentation rate (mm/h)	48 (27–67) *N* = 17	79 (44–98) *N* = 4	47 (27–63) *N* = 13	0.23
Ferritin (ng/ml)	751 (270–2560) *N* = 46	1052 (270–2647) *N* = 11	704 (255–2293) *N* = 35	0.53
Interleukin-6 (pg/ml)	37.0 (7.7–60.0) *N* = 9	1056 (540–1572) *N* = 2	33.0 (5.0–56.0) *N* = 7	0.09
Procalcitonin (ng/ml)	0.64 (0.18–4.22) *N* = 49	2.77 (0.75–10.69) *N* = 12	0.40 (0.15–2.18) *N* = 37	0.10
Markers of organ dysfunction^c^ – *n* (%)				
Elevated cardiac troponin^b^	31 (68.9) *N* = 45	6 (66.7) *N* = 9	25 (69.4) *N* = 36	1.00
Elevated BNP or NT-proBNP	26 (78.8) *N* = 33	6 (85.7) *N* = 7	20 (76.9) *N* = 26	1.00
Elevated creatinine	28 (52.8) *N* = 53	5 (41.7) *N* = 12	23 (56.1) *N* = 41	0.51
Elevated alanine aminotransferase	21 (41.2) *N* = 51	7 (58.3) *N* = 12	14 (35.9) *N* = 39	0.20

IQR, interquartile range; BNP, B-type natriuretic peptide; NT-proBNP, N-terminal proB-type natriuretic peptide.

a Thresholds for blood cell counts: Leucocytosis = absolute lymphocyte count >11.0 K/μl, Neutrophilia = absolute neutrophil count >8.0 K/μl, Lymphopenia = absolute lymphocyte count <1.0 K/μl, Anaemia = haemoglobin <14.0 mg/dl (men), <12.0 mg/dl (women), Thrombocytopenia = platelets <150 K/μl.

b Includes both troponin-I and troponin-T. Troponin values were classified as elevated or normal based on the reference range used by the testing hospital laboratory.

c Thresholds for markers of organ dysfunction: Elevated B-type natriuretic peptide = >100 pg/ml, Elevated N-terminal proB-type natriuretic peptide = >450 pg/ml, Elevated creatinine = >1.3 mg/dl, Elevated alanine aminotransferase = >55 IU/l.

**Table 4.** Treatments administered to patients meeting modified criteria for multisystem inflammatory syndrome in adults with discharge during March–December 2020 in the Premier Healthcare Database Special COVID-19 Release

	All patients *N* = 53	Patients <50 years-old *N* = 12	Patients ⩾50 years-old *N* = 41	*P*-value
Cardiovascular support – *n* (%)				
Vasoactive medications	31 (58.5)	7 (58.3)	24 (58.5)	1.00
Intra-aortic balloon pump	2 (3.8)	2 (16.7)	0 (0.0)	<0.05
Respiratory support – *n* (%)				
Mechanical ventilation	30 (56.6)	7 (58.3)	23 (56.1)	1.00
Veno-venous ECMO	1 (1.9)	1 (8.3)	0 (0.0)	0.23
Medications administered – *n* (%)				
Intravenous corticosteroids	39 (73.6)	10 (83.3)	29 (70.7)	0.48
Intravenous immunoglobin	1 (1.9)	1 (8.3)	0 (0.0)	0.23
Tocilizumab	18 (34.0)	5 (41.7)	13 (31.7)	0.73

ECMO, extracorporeal membrane oxygenation.

### Demographic, clinical, and laboratory characteristics among patients under 50 years of age

Among 12 patients in the potential MIS-A cohort less than 50 years-old, the median age was 41 years (IQR 32–44) (Table 1). Seventy-five per cent of patients were male. The most common racial/ethnic group was non-Hispanic black (33.3%), followed by Hispanic (25.0%), non-Hispanic white (25.0%), Asian (8.3%), and unknown race (8.3%). Although the proportion of non-Hispanic black and Hispanic patients was higher among those aged less than 50 years than among those over 50 years, the racial/ethnic composition of the cohort did not differ significantly between the age groups (*P* = 0.15).

Hypertension was less common among patients under 50 than among those aged greater than or equal to 50 years (16.7% *vs.* 58.5%, *P* = 0.02) (Table 1). The proportion with other chronic medical conditions did not differ significantly. Similarly, no significant differences were found in the clinical criteria, length of stay, ICU admission, and the proportion who died across age groups (Table 2).

As in the full cohort, laboratory abnormalities were common among patients aged less than 50 years (Table 3). The proportion of patients with leucocytosis (*P* = 0.02) and neutrophilia (*P* = 0.03) were higher among patients less than 50 years old than over 50 years old. Median values for all inflammatory markers appeared higher in the younger age group than the older age group, although only one of these comparisons was statistically significant (CRP: *P* = 0.03).

The proportion of patients who received vasoactive medications, mechanical ventilation, intravenous corticosteroids, IVIG, and tocilizumab did not differ significantly between patients younger and older than 50 years of age (Table 4). However, both of the patients in the cohort who received intra-aortic balloon pumps were under 50 years of age, making this treatment more common in the younger age group (*P* < 0.05).

## Discussion

To assess how commonly MIS-A occurs among patients hospitalised with SARS-CoV-2-related illness and to describe the clinical characteristics of this condition, we applied criteria adapted from the CDC case definition for MIS-A to the PHD-SR. Our results suggest that MIS-A is a very rare outcome of SARS-CoV-2 infection. Out of 34 515 hospitalisations with complete laboratory data to which our criteria could be applied, only 53 (0.15%) met the criteria for potential MIS-A. These results are somewhat consistent with a recent study by Davogustto *et al*. in which MIS-A was also found to be a rare outcome among SARS-CoV-2-related hospitalisations [17]. In that study, of 839 patients admitted with laboratory evidence of SARS-CoV-2 infection, 15 (1.8%) met the criteria for MIS-A. Although the proportion of hospitalisations meeting MIS-A criteria was higher in their analysis than in ours, this discrepancy is likely explained by differences in methodology. While our study used MIS-A criteria adapted from the CDC case definition, the Davogustto *et al*. study was conducted before the CDC case definition was published. Therefore, the investigators used MIS-A criteria that differed from our criteria in a number of important ways. For example, in Davogustto *et al*., only 8 of the 15 patients classified as MIS-A had cardiovascular involvement that could have met MIS-A criteria in our study. In addition, cardiovascular involvement was defined as including several conditions that would not have met our severe cardiac illness criterion (e.g. hypotension, elevated troponin), indicating that the number of patients in Davogustto *et al*. who would have met our modified MIS-A criteria is even lower. Future research should use the CDC case definition for identifying MIS-A to facilitate the comparison of results across studies.

Our results also reveal additional information about the clinical and treatment characteristics of patients with MIS-A. The most common cardiac manifestations detected in the potential MIS-A cohort were myocarditis (66.0%) and new-onset heart failure (35.9%), while pericarditis (5.7%) and coronary artery aneurysm (1.9%) were less common. In contrast, a study of 1733 MIS-C patients reported to CDC from March 2020–January 2021 found that 17% of patients had myocarditis, 31% had cardiac dysfunction, and 17% had coronary artery aneurysm or dilatation [18]. These results suggest that myocarditis may be more common, and coronary artery aneurysm more rare, in MIS-A compared to MIS-C. Most patients in the potential MIS-A cohort met at least two clinical criteria through a combination of severe cardiac illness and either shock/hypotension or thrombocytopenia. Few patients had gastrointestinal involvement and no patients were found to have rash or conjunctivitis. The lack of patients in the potential MIS-A cohort with gastrointestinal symptoms, rash and conjunctivitis is likely due to underreporting of these manifestations with ICD-10-CM diagnosis codes. Existing data on MIS-A and MIS-C indicate that both gastrointestinal symptoms and mucocutaneous manifestations are important elements of these syndromes [5, 8, 19, 20]. A case-based review of published reports describing MIS-A found that 72.5% of patients had gastrointestinal symptoms, 39.2% had dermatologic findings, and 35.3% had conjunctival findings [5]. Interestingly, only one patient in the potential MIS-A cohort received IVIG. This finding may be attributable to several factors, including the fact that MIS-A is vastly underdiagnosed and no guidelines have been developed for its treatment [17]. Finally, outcome data from the potential MIS-A cohort are also revealing. Most patients required ICU admission and 43% died. The proportion who died is inconsistent with the published literature on MIS-A and MIS-C, which indicates that deaths from these syndromes are rare [5, 8, 18, 19]. However, it is possible that the criteria used to define the potential MIS-A cohort were more likely to identify patients with the most severe presentations of the disease.

Alternatively, the large proportion of deaths in the cohort may be explained by the inclusion of patients with hyperinflammatory phenotypes other than MIS-A. The demographic and clinical characteristics of our cohort suggest that many patients who met modified MIS-A criteria may alternatively have had severe COVID-19. As mentioned above, MIS-A case reports often describe patients under 50 years of age with few comorbidities [4, 5]. By contrast, the median age in our cohort was 62 years (IQR 52–74) with 77% of patients over age 50. More than 40% of patients in the cohort had a history of either hypertension or diabetes, and the proportion of patients who died was quite high, as discussed above. These characteristics combined with the finding that nearly all cohort patients had respiratory involvement of their disease and 57% required mechanical ventilation suggest that some of the cohort may be comprised of patients with severe COVID-19. Distinguishing MIS-A from severe acute COVID-19 is especially difficult among adults due to their high prevalence of comorbidities and more severe COVID-19 illness in comparison to children [16]. Disentangling these conditions is further complicated by the fact that they have many signs and symptoms in common. Fever, myocarditis, heart failure, gastrointestinal symptoms, and elevated inflammatory markers can all occur in the setting of either MIS-A or severe COVID-19. Additional studies comparing clinical and laboratory features of MIS-A and severe COVID-19 are needed to improve our understanding of how these conditions differ.

Our analysis has several limitations. Because complete laboratory data on inflammatory markers were only available for 9.0% of hospitalisations in the PHD-SR, our modified criteria for MIS-A could only be applied to this subset of hospitalisations. Analysis of ICU admissions and deaths among hospitalisations with and without available laboratory data revealed no obvious differences in illness severity between these groups, but whether other differences exist is unclear. Furthermore, SARS-CoV-2-related hospitalisations were identified using ICD-10-CM codes for coronavirus and COVID-19; however, given that RT-PCR testing for SARS-CoV-2 may be negative in MIS-A, and serology testing is not routinely ordered on all patients, some MIS-A hospitalisations may not have received a diagnosis code for coronavirus and would not have been identified by our criteria as a result. ICD-10-CM codes used to define certain clinical criteria may also have been poor indicators of whether these signs and symptoms were present, resulting in missed MIS-A cases. Finally, because our analysis was based on administrative discharge data as opposed to medical chart review, it was not possible to perform a clinical assessment of each hospitalisation. Individual case review may have revealed more likely alternative diagnoses, and some hospitalisations in the cohort may not represent MIS-A, as discussed above. However, despite the limitations of the sensitivity and specificity of our criteria, our results still suggest that MIS-A is a rare outcome of SARS-CoV-2 infection. Given that 0.15% of hospitalisations met the criteria for potential MIS-A, even if the sensitivity of our criteria were only 10%, the true frequency of MIS-A among SARS-CoV-2-related hospitalisations would still be low at 1.5%.

MIS-A has been recognised as an outcome of SARS-CoV-2 infection, but its epidemiology remains poorly understood. This study represents one of the first analyses to examine the frequency of MIS-A among hospitalised patients and to describe their characteristics. Our results suggest that MIS-A is a rare but clinically severe syndrome with features that may overlap with severe COVID-19 in adults. Further studies should examine differences between MIS-A and severe COVID-19 to better understand their epidemiology and to facilitate diagnosis. Increasing awareness of MIS-A will facilitate a greater understanding of this condition and help quantify the true burden of disease. Public health and healthcare partners should collaborate to educate providers on how to recognise and report cases of MIS-A when they occur.

## Data Availability

The data that support the findings of this study are from the Premier Healthcare Database Special COVID-19 Release, a commercially available database that cannot be made public, but is available for purchase.

## Supplementary material

For supplementary material accompanying this paper visit https://doi.org/10.1017/S0950268822000024. click here to view supplementary material
