# Supplementary material for: Identification and description of patients with multisystem inflammatory syndrome in adults associated with SARS-CoV-2 infection using the Premier Healthcare Database
Source: Epidemiol Infect. 2022 Jan 17;150:e26. doi: 10.1017/S0950268822000024 (PMC8886080; doi:10.1017/S0950268822000024)
Supplement: Supplementary file 1 [file S0950268822000024sup001.docx]

Journal: *Epidemiology and Infection*

Title: Identification and description of patients with multisystem inflammatory syndrome in adults associated with SARS-CoV-2 infection using the Premier Healthcare Database

Authors: Jennifer DeCuir, James Baggs, Michael Melgar, Pragna Patel, Karen K. Wong, Noah G. Schwartz, Sapna Bamrah Morris, Shana Godfred-Cato, Ermias D. Belay

**SUPPLEMENTARY MATERIAL**

**CRITERIA USED TO DEFINE THE POTENTIAL MIS-A COHORT**

INCLUSION CRITERIA

1. Age ≥ 21 years old
2. Confirmed SARS-CoV-2-related hospitalization

- For admissions in Feb-April 2020 and discharges in March-April 2020, SARS-CoV-2-related hospitalization was defined as a primary or secondary ICD-10 code of B97.29
- For discharges from April 2020 on, SARS-CoV-2-related hospitalization was defined as a primary or secondary ICD-10 code of U07.1
- Note: Hospitalizations in which these codes were present on admission (i.e. ICD_PRI_SEC = “A”) were not included.

1. Laboratory evidence of severe inflammation as evidenced by ≥2 elevated inflammatory markers within the first 3 days of hospitalization (Hospital day 0-3):

- IL-6 > 2.0 pg/mL
- CRP > 1 mg/dL
- Ferritin > 400 ng/mL
- ESR > 20 mm/hr
- Procalcitonin > 0.1 ng/mL

1. Severe cardiac illness AND ≥ 1 additional clinical criterion:
   - Severe cardiac illness

- New-onset heart failure
  - I50.1 Left ventricular failure, unspecified
  - I50.20 Unspecified systolic (congestive) heart failure
  - I50.21 Acute systolic (congestive) heart failure
  - I50.40 Unspecified combined systolic (congestive) and diastolic (congestive) heart failure
  - I50.41 Acute combined systolic (congestive) and diastolic (congestive) heart failure
  - I50.82 Biventricular heart failure
- Myocarditis
  - B33.22 – Viral myocarditis
  - I40.0 – Infective myocarditis
  - I40.8 – Other acute myocarditis
  - I40.9 – Acute myocarditis, unspecified
  - I51.4 – Myocarditis, unspecified
- Pericarditis
  - I30.0 – Acute nonspecific idiopathic pericarditis
  - I30.1 – Infective pericarditis
  - I30.8 – Other forms of acute pericarditis
  - I30.9 – Acute pericarditis, unspecified
- Coronary artery aneurysm
  - I25.41 – Coronary artery aneurysm
- Mucocutaneous manifestations
  - Rash
    - R21 – Rash and other non-specific skin eruption
  - Non-purulent conjunctivitis
    - B30.8 – Other viral conjunctivitis
    - B30.9 – Viral conjunctivitis, unspecified
    - H10.30 – Unspecified acute conjunctivitis, unspecified eye
    - H10.33 – Unspecified acute conjunctivitis, bilateral
    - H10.89 – Other conjunctivitis
    - H10.9 – Unspecified conjunctivitis
- Shock or hypotension
  - Treated with vasoactive agent on ≥2 different days at any time during hospitalization
    - Dobutamine
    - Dopamine
    - Ephedrine
    - Epinephrine
    - Isoproterenol
    - Milrinone
    - Nitroprusside
    - Norepinephrine
    - Phenylephrine
    - Vasopressin
  - Shock
    - R57.0 – Cardiogenic shock
    - R57.8 – Other shock
    - R57.9 – Shock, unspecified
    - R65.21 – Severe sepsis with septic shock
  - Hypotension
    - I95.0 – Idiopathic hypotension
    - I95.89 – Other hypotension
    - I95.9 – Hypotension, unspecified
  - R65.20 – Severe sepsis without septic shock
- Abdominal pain, vomiting, or diarrhea
  - Abdominal pain
    - R10.10 - Upper abdominal pain, unspecified
    - R10.30 - Lower abdominal pain, unspecified
    - R10.84 - Generalized abdominal pain
    - R10.9 - Unspecified abdominal pain
  - Vomiting
    - R11.11 - Vomiting without nausea
    - R11.2 – Nausea with vomiting, unspecified
    - R11.10 – Vomiting unspecified
  - Diarrhea
    - R19.7 – Diarrhea, unspecified
  - Gastroenteritis
    - A09 - Infectious gastroenteritis and colitis, unspecified
    - A08.39 – Other viral enteritis
    - A08.4 – Viral intestinal infection, unspecified
- Thrombocytopenia
- D69.6 – Thrombocytopenia, unspecified
- D69.59 – other secondary thrombocytopenia
- Platelet count <150 K/uL

EXCLUSION CRITERIA

- History of chronic heart failure at baseline
  - Patients were considered to have heart failure at baseline if they had ≥1 of the following:
    - A history of congestive heart failure (CHF) according to the Elixhauser algorithm.
      - For the purposes of identifying patients with CHF, the Elixhauser algorithm was applied to hospital encounter data from the Premier Healthcare Database dating from January 1, 2019 up to (but not including) the first COVID hospitalization. Patients with a heart failure ICD code on a previous hospital encounter as defined by the Elixhauser algorithm were classified as having heart failure at baseline.
    - An ICD code from the COVID hospitalization indicative of heart failure at baseline
      - I11.0 – Hypertensive heart disease with heart failure
      - I13.0 – Hypertensive heart and chronic kidney disease with heart failure and stage 1 through stage 4 chronic kidney disease, or unspecified chronic kidney disease
      - I13.2 – Hypertensive heart and chronic kidney disease with heart failure and with stage 5 chronic kidney disease, or end stage renal disease
      - I27.81 – Cor pulmonale (chronic)
      - I50.22 – Chronic systolic (congestive) heart failure
      - I50.23 – Acute on chronic systolic (congestive) heart failure
      - I50.32 – Chronic diastolic (congestive) heart failure
      - I50.33 – Acute on chronic diastolic (congestive) heart failure
      - I50.42 – Chronic combined systolic (congestive) and diastolic (congestive) heart failure
      - I50.43 – Acute on chronic combined systolic (congestive) and diastolic (congestive) heart failure
      - I50.811 – Chronic right heart failure
      - I50.812 – Acute on chronic right heart failure
- Alternative diagnosis for new-onset heart failure
  - I21.01 – ST elevation (STEMI) myocardial infarction involving left main coronary artery
  - I21.02 – ST elevation (STEMI) myocardial infarction involving left anterior descending coronary artery
  - I21.09 – ST elevation (STEMI) myocardial infarction involving other coronary artery of anterior wall
  - I21.11 – ST elevation (STEMI) myocardial infarction involving right coronary artery
  - I21.19 – ST elevation (STEMI) myocardial infarction involving other coronary artery of inferior wall
  - I21.21 – ST elevation (STEMI) myocardial infarction involving left circumflex coronary artery
  - I21.29 – ST elevation (STEMI) myocardial infarction involving other sites
  - I21.3 – ST elevation (STEMI) myocardial infarction of unspecified site
  - I25.5 – Ischemic cardiomyopathy
  - I42.1 – Obstructive hypertrophic cardiomyopathy
  - I42.2 – Other hypertrophic cardiomyopathy
- Sepsis due to an alternative pathogen
  - A40.0 – Sepsis due to streptococcus, group A
  - A40.1 – Sepsis due to streptococcus, group B
  - A40.3 – Sepsis due to Streptococcus pneumoniae
  - A40.8 – Other streptococcal sepsis
  - A40.9 – Streptococcal sepsis, unspecified
  - A41.01 – Sepsis due to Methicillin susceptible Staphylococcus aureus
  - A41.02 – Sepsis due to Methicillin resistant Staphylococcus aureus
  - A41.1 – Sepsis due to other specified staphylococcus
  - A41.2 – Sepsis due to unspecified staphylococcus
  - A41.3 – Sepsis due to Hemophilus influenzae
  - A41.4 – Sepsis due to anaerobes
  - A41.50 – Gram-negative sepsis, unspecified
  - A41.51 – Sepsis due to Escherichia coli [E. coli]
  - A41.52 – Sepsis due to Pseudomonas
  - A41.53 – Sepsis due to Serratia
  - A41.59 – Other Gram-negative sepsis
  - A41.81 – Sepsis due to Enterococcus
  - B37.7 – Candidal sepsis
  - R78.81 – Bacteremia
  - T80.211A – Bloodstream infection due to central venous catheter

**OTHER VARIABLE DEFINITIONS**

**Definition of Respiratory Involvement**

- Hospitalizations with ≥ 1 of the ICD codes below were classified as having respiratory involvement of their SARS-CoV-2 infection

| **ICD 10-CM Code** | **ICD Code Description** |
| --- | --- |
| J12.81 | Pneumonia due to SARS-associated coronavirus |
| J12.82 | Pneumonia due to coronavirus disease 2019 |
| J12.89 | Other viral pneumonia |
| J12.9 | Viral pneumonia, unspecified |
| J18.8 | Other pneumonia, unspecified organism |
| J18.9 | Pneumonia, unspecified organism |
| J80 | Acute respiratory distress syndrome |

**Definitions of Mechanical Circulatory Support Variables**

**Intra-aortic balloon pump**

- Hospitalizations with ≥ 1 of the ICD codes below were classified as receiving an intra-aortic balloon pump

| **ICD-10-PCS Code** | **ICD Code Description** |
| --- | --- |
| 5A02110 | Assistance with Cardiac Output using Balloon Pump, Intermittent |
| 5A02210 | Assistance with Cardiac Output using Balloon Pump, Continuous |

**Ventricular assist device**

- Includes both implanted ventricular assist devices and percutaneous ventricular assist devices (e.g. TandemHeart, Impella)
- Hospitalizations with ≥ 1 of the ICD codes below were classified as receiving a ventricular assist device

| **ICD-10-PCS Code** | **ICD Code Description** |
| --- | --- |
| 02HA0QZ | Insertion of Implantable Heart Assist System into Heart, Open Approach |
| 02HA0RS | Insertion of Biventricular Short-term External Heart Assist System into Heart, Open Approach |
| 02HA0RZ | Insertion of Short-term External Heart Assist System into Heart, Open Approach |
| 02HA3QZ | Insertion of Implantable Heart Assist System into Heart, Percutaneous Approach |
| 02HA3RS | Insertion of Biventricular Short-term External Heart Assist System into Heart, Percutaneous Approach |
| 02HA3RZ | Insertion of Short-term External Heart Assist System into Heart, Percutaneous Approach |
| 02HA4QZ | Insertion of Implantable Heart Assist System into Heart, Percutaneous Endoscopic Approach |
| 02HA4RS | Insertion of Biventricular Short-term External Heart Assist System into Heart, Percutaneous Endoscopic Approach |
| 02HA4RZ | Insertion of Short-term External Heart Assist System into Heart, Percutaneous Endoscopic Approach |
| 5A02116 | Assistance with Cardiac Output using Other Pump, Intermittent |
| 5A02216 | Assistance with Cardiac Output using Other Pump, Continuous |
| 5A0211D | Assistance with Cardiac Output using Impeller Pump, Intermittent |
| 5A0221D | Assistance with Cardiac Output using Impeller Pump, Continuous |

**VA-ECMO**

- Hospitalizations with ≥ 1 of the ICD codes below were classified as receiving VA-ECMO

| **ICD-10-PCS Code** | **ICD Code Description** |
| --- | --- |
| 5A1522F | Extracorporeal Oxygenation, Membrane, Central |
| 5A1522G | Extracorporeal Oxygenation, Membrane, Peripheral Veno-arterial |

**VV-ECMO**

- Hospitalizations with ≥ 1 of the ICD codes below were classified as receiving a VV-ECMO

| **ICD-10-PCS Code** | **ICD Code Description** |
| --- | --- |
| 5A1522H | Extracorporeal Oxygenation, Membrane, Peripheral Veno-venous |
